# miRNA Profiles in Patients with Hematological Malignancy at Different Stages of the Disease: A Preliminary Study

**DOI:** 10.3390/biomedicines12081924

**Published:** 2024-08-22

**Authors:** Jood Hashem, Lujain Alkhalaileh, Hassan Abushukair, Mahmoud Ayesh

**Affiliations:** 1 Department of Medical Laboratory Sciences, Jordan University of Science and Technology, Irbid 22110, Jordan; ljalkhalaileh21@ams.just.edu.jo; 2 Faculty of Medicine, Jordan University of Science and Technology, Irbid 22110, Jordan; hmabushukair182@med.just.edu.jo (H.A.); mhhajyousef@just.edu.jo (M.A.)

**Keywords:** miRNAs, newly diagnosed, remission, resistance, hematological malignancies

## Abstract

The dysregulation of miRNA expression has been shown to impact cellular physiology and tumorigenesis. Studies have reported several miRNA regulatory elements and pathways that play a significant role in the diagnosis, prognosis, and treatment of hematological malignancies. This is the first study to test the differential expression of miRNAs at crucial stages of the disease, specifically newly diagnosed, resistant to treatment, and remission. Circulating miRNAs extracted from the blood samples of 18 patients diagnosed with leukemia or lymphoma at different stages and 2 healthy controls were quantified by qPCR using a panel of 96 tumorigenic miRNAs. An enrichment analysis was performed to understand the mechanisms through which differential miRNA expression affects cellular and molecular functions. Significant upregulation of hsa-miR-1, hsa-miR-20a-5p, hsa-miR-23a-3p, hsa-miR-92b3p, and hsa-miR-196a-5p was detected among the different stages of leukemia and lymphoma. mir-1 and mir-196a-5p were upregulated in the remission stage of leukemia, while mir-20a-5p, mir-23a-3p, and mir-92b-3p were upregulated during the resistant stage of lymphoma. The enrichment analysis revealed these miRNAs’ involvement in the RAS signaling pathway, TGF-β signaling, and apoptotic pathways, among others. This study highlights new biomarkers that could be used as potential targets for disease diagnosis, prognosis, and treatment, therefore enhancing personalized treatments and survival outcomes for patients.

## 1. Background

Hematological malignancies (HMs) account for one-fifth of all cancers and are the second leading cause of cancer deaths [1]. HMs are cancers that affect the bone marrow, blood, and lymph nodes [2]. Depending on the type of cell affected, they are characterized as leukemia, lymphoma, or myeloma [3]. Genomic instability is one of the main causes of HMs and is mainly connected to both hereditary and acquired leukemias [4]. Genomic instability results from a variety of pathways, including centrosome amplification, telomere damage, epigenetic alterations, and DNA damage from endogenous and external causes. It can be perpetuated or limited by the generation of mutations or aneuploidy [5]. Currently, the molecular pathogenesis of HMs includes the activation of oncogenes, the inactivation of tumor suppressor genes, or the blocking of differentiation [6,7,8].

There is a crucial role for signaling pathways such as the Wnt, PI3K/AKT, NOTCH, TGF-beta, NF-κB, and JAK/STAT pathways in the development of cancers, including HMs [9]. In particular, in myeloid cancers, like acute myeloid leukemia (AML) and myelodysplastic syndrome (MDS), the RAS pathway is often dysregulated, resulting in persistent growth signals [10,11,12,13]. TGF-β can act as both a tumor suppressor and a promoter, aiding in processes like apoptosis or metastasis [14,15]. In leukemia, the hyperactivation of the MAPK pathway, often caused by mutations to RAS or RAF, contributes to increased cell survival and proliferation [16,17]. Research is ongoing to develop small molecule inhibitors targeting these pathways.

Only about 1% of DNA contains protein-coding regions, while the remaining 99% is noncoding [18]. These noncoding regions include miRNAs, long noncoding RNAs (lncRNAs), and circular RNAs (circRNAs). They are involved in cell differentiation, cell growth, and proliferation and play a significant role in human disease [19,20,21]. HMs are characterized by the molecular dysregulation of miRNA expression that can affect the expression of genes associated with hematopoiesis, cell cycle control, apoptosis, angiogenesis, and the immune response [22].

miRNAs control the translation of 20–30% of all human genes [23]. miRNAs can be used to assess the likelihood of disease progression or relapse and identify patients who are likely to respond to specific treatments or generate resistance to drugs making them valuable for personalized treatment plans [24,25,26,27,28]. Increased levels of circulating miR-181b-5p and miR-155-3p were found in the blood of AML patients, and miR-181-5p expression was found to be linked to a shorter overall survival [29]. Let-7f, miR-9, and miR-27a are examples of miRNAs that can be used to differentiate classical Hodgkin lymphoma from other B-cell lymphoma cell lines [30]. Furthermore, circulating miRNAs were found to be correlated with the stage of cancer, i.e., miRNA-141 was correlated with stage IV colon cancer, and miRNA-21 was correlated with metastases of colorectal cancer [31,32,33]. These results, including others, suggest the use of miRNAs as biomarkers for the evaluation of cancer risk and its progression.

Studies have revealed that different classes of miRNAs have differential functions depending on their tissue localization and target [34]. They can be tumor-suppressive or oncogenic in HMs according to different studies [35,36,37]. However, these studies have traditionally focused on the expression of miRNAs in HMs in general without looking into the expression of a panel of miRNAs at different stages of the disease. To the best of our knowledge, this study is the first of its kind to look into the relationship between miRNA expression levels in reference to the type and stage of the disease. Thus, to tackle the current research problem, this study examined the miRNA profiles of patients with HMs at different stages of the disease (newly diagnosed (ND), in remission (Rem), and resistant to treatment (Res)).

## 2. Methods

### 2.1. Subjects and Selection Criteria

A case–control study was conducted from March 2023 to December 2023, collecting 18 blood samples from patients who visited the King Abdullah University Hospital’s (KAUH) hematology clinic. In total, 9 samples were obtained from leukemia patients and another 9 samples were obtained from lymphoma patients at different stages of the disease. The stages were characterized as ND, Rem, and Res. For each stage, a total of 3 samples were analyzed. Additionally, 2 samples were collected from healthy control patients. The stages were classified by the patients’ primary physician, a hematology oncologist, based on the following conditions:

ND: A patient who is newly diagnosed with the disease (leukemia/lymphoma) but has not started any form of treatment. Samples were taken at diagnosis.
Rem: A leukemia/lymphoma patient who received a course of treatment and their medical tests show no signs of the disease.
Res: A leukemia/lymphoma patient who received treatment but failed to show any signs of improvement. Some patients who provided blood samples eventually passed away months later.

The inclusion criteria for this study included patients with no history of any other diseases for the control group and those suffering from leukemia or lymphoma and have not been diagnosed with any other type of malignancy. Patients with a history of other types of malignancy were excluded. Ethical approval was obtained from the Institutional Review Board (IRB) committee at KAUH and the Jordan University of Science and Technology before collecting the samples (155/2023). Written informed consent was obtained from each participant before their enrollment in the study.

### 2.2. Sample Collection, Handling, and Transport

Blood samples were collected by venipuncture from all participants. About 8 mL of blood was collected in Streck tubes (Cat# 346064-6 Streck Co., La Vista, NE, USA) The samples were transported within 2 h in an icebox to the laboratory for immediate processing. The blood samples were centrifuged for 20 min at 3200 rpm to extract the plasma. The plasma was stored at −80 °C until further analysis.

### 2.3. Molecular Studies

#### 2.3.1. miRNA Extraction and cDNA Synthesis

An miRNeasy Serum/Plasma kit (Cat# 217184 Qiagen, Hilden, Germany) was used to extract miRNAs from plasma. The procedure was performed using standard phenol/guanidine lysis of the samples and silica-membrane-based purification using RNeasy MinElute spin columns [38]. Complementary DNA (cDNA) was synthesized using a miRCURY LNA RT Kit for 8–64 cDNA synthesis reactions (Cat# 339340 Qiagen, Germany).

#### 2.3.2. miRNA Quantification by Real-Time qPCR

Circulating miRNAs were quantified after cDNA synthesis by real-time quantitative PCR (rt-qPCR) [39] using QuantStudio Real-Time PCR. The quantitative assay was performed using a panel of 96 miRNAs involved in tumorigenesis, apoptosis, and differentiation pathways from the Qiagen miRCURY LNA miRNA Focus Panel—Cancer (Cat# 339325 Qiagen, Germany), as shown in Supplementary Table S1. This panel included three snRNA reference genes (U6snRNA, SNORD38B, and SNORD49A), three inter-plate calibrators (UniSp3 IPC), five RNA spike-ins (cel-miR-39-3p, UniSp2, UniSp4, UniSp5, and UniSp6), a no template control (blank H2O), and five miRNA reference genes for normalization (miR-103a-3p, miR-191-5p, miR-423-5p, let-7a-5p, and miR-16-5p). Following this, 79 miRNAs were left for analysis in this study.

The cycle threshold (CT) was set within the exponential phase of the amplification plots. The relative difference in expression levels between the control and patient samples was determined by comparing cycle threshold CT values (2^−∆∆CT^).

### 2.4. Statistical Analysis

#### 2.4.1. Fold Change Calculation

To analyze the differential expression of miRNAs between the different stages, the fold change was calculated using the Livak method (2^−∆∆CT^) for relative quantification [40]. The ∆CT for each miRNA was calculated by subtracting the CT value of the housekeeping genes from the CT value of the miRNA of interest. The ∆∆Ct was then calculated by subtracting the ∆CT of the control group from the ∆CT of the comparison group.

#### 2.4.2. Statistical Testing

To determine the statistical significance of the observed fold changes, Mann–Whitney U tests were performed using Jamovi statistical analysis software to compare the ∆CT values of each miRNA between the different stages. The Mann–Whitney U test is a non-parametric test that does not assume a normal distribution of the data, making it suitable for analyzing qPCR results [41].

A *p*-value < 0.1 was considered statistically significant due to the small sample size and the exploratory nature of our study. The severity of HMs, combined with the challenges of recruiting patients who often face significant health issues, limited our sample size to 18 patients. Additionally, budget and time constraints precluded the possibility of expanding the sample size. In early-stage, exploratory research, a more lenient *p*-value can help balance the risk of false positives with the risk of missing potentially relevant findings. While a *p*-value of 0.05 is commonly used in confirmatory studies, it may be overly stringent in an exploratory context, as it can lead to overlooking important associations [42]. Therefore, a significance level of 0.1 increases the sensitivity of the analysis to detect possible associations, particularly in the context of biomarker usage in diagnosis and prognosis.

### 2.5. Bioinformatics Analysis

To further understand the underlying molecular functions and mechanisms involved in the differential enrichment of miRNAs across different patient groups (ND, Rem, and Res), potential target genes of differentially expressed miRNAs were identified for each miRNA using miRWalk2.0. The selection criteria for the target genes included those that were validated and found to correlate with the miRNA in the TargetScan or miRDB database (June 2024 release). A gene ontology (GO) analysis (http://geneontology.org, accessed on 20 June 2024) was performed for the target genes at three levels: molecular function (MF), biological process (BP), and cellular component (CC). A pathway analysis was performed using the Kyoto Encyclopedia of Genes and Genomes database (KEGG) (www.genome.jp/kegg, accessed on 20 June 2024). Functional enrichment and pathway enrichment analyses were performed by using the Database for Annotation, Visualization, and Integrated Discovery (DAVID) web tool (http://david-d.ncifcrf.gov accessed on 20 June 2024). Both GO and KEGG analyses were conducted for the target genes using the DAVID 6.8 bioinformatics tool (*p*-value < 0.05). Heatmaps and bubble plots were generated using the SRplot online platform, a free online platform for data visualization and graphing [43].

## 3. Results

### 3.1. Patients’ Characteristics

A total of 18 patients with HMs were enrolled in the study. Those included 9 leukemia patients and 9 lymphoma patients. Samples were obtained from 3 different stages of the disease: ND, Rem, and Res stages. Each of the three stages was represented by three of the enrolled patients. In addition, two healthy controls were enrolled in the study. Detailed patient information is represented in Supplementary Table S2.

### 3.2. Overview of miRNA Expression across Disease Stages

**Figure 1:**
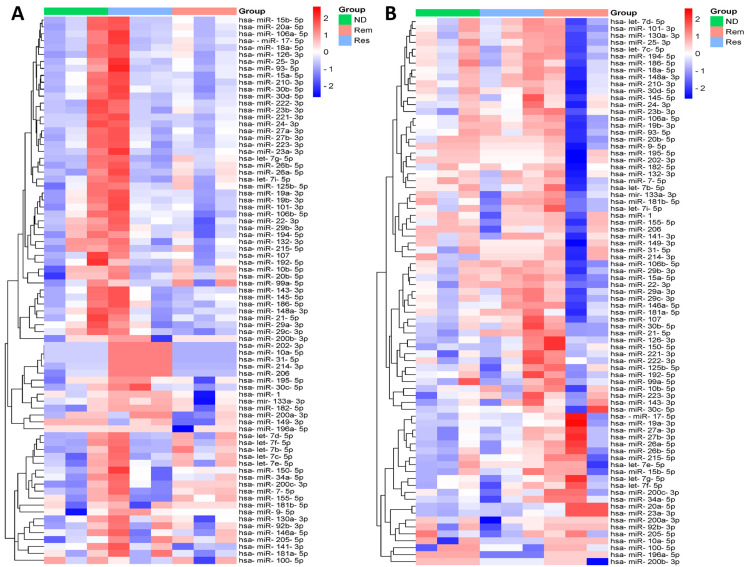
Heatmap showing hierarchical clustering of differential expression patterns of miRNAs between different stages in (**A**) leukemia and (**B**) lymphoma samples.

To visualize the expression patterns of all miRNAs across the three stages of leukemia and lymphoma separately, two clustered heatmaps containing 79 miRNAs were constructed, as shown in Figure 1. Each row in the heatmap represents a single miRNA and each column represents a specific stage of the disease, including ND (green), Rem (light red), and Res (light blue). The color scale bar on the right, ranging from blue to red, represents the relative expression of the miRNAs, with blue indicating downregulation and red indicating upregulation. Each heatmap revealed distinct expression patterns among the different stages of the disease, with clusters of miRNAs showing hierarchical upregulation or downregulation across the patient samples. In particular, several miRNAs displayed stage-specific expression patterns, suggesting their potential roles in determining/predicting disease progression, treatment follow-up, and response.

### 3.3. miRNA Differential Expression in Newly Diagnosed Leukemia and Lymphoma Patients vs. Healthy Controls

Differential miRNA expression was assessed in ND leukemia and lymphoma patients and compared to healthy controls. Out of 79 miRNAs, 5 miRNAs were upregulated in leukemia and 11 miRNAs were upregulated in lymphoma (Supplementary Table S3). However, due to their relevance according to the literature and their high fold change, two miRNAs were chosen to be presented in Table 1. The fold change calculations in Table 1 indicate that miR-223-3p was upregulated in ND leukemia patients, while miR-24-3p was upregulated in ND lymphoma patients.

### 3.4. Comparative Analysis for the Differential Expression of 5 miRNAs in Leukemia and Lymphoma

**Figure 2:**
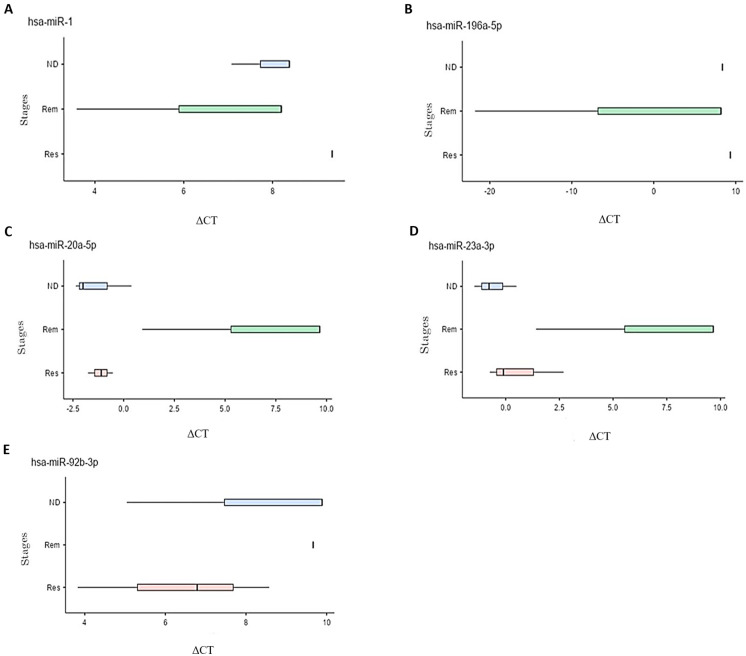
Box plots representing the different ΔCT values at ND, Rem, and Res stages for multiple differentially expressed miRNAs in leukemia and lymphoma samples. Significance was defined as a fold change ≥ 2 or ≤0.5 with a *p*-value < 0.1. Five miRNAs met these criteria, all of them upregulated. (**A**) Upregulation of mir-1 in the Rem stage compared to the ND and Res stages in patients with leukemia. (**B**) Upregulation of mir-196a-5p in the Rem stage compared to the ND and Res stages in patients with leukemia. (**C**) Upregulation of mir-20a-5p in the Res stage compared to the ND and Rem stages in patients with lymphoma. (**D**) Upregulation of mir-23a-3p in the Res stage compared to the Rem stage and upregulation in the ND stage compared to the Rem and Res stages in patients with lymphoma. (**E**) Upregulation of mir-92b-3p in the Res stage compared to the ND and Rem stages and an upregulation in the ND compared to Rem stage in patients with lymphoma.

The differential miRNA expression was subsequently tested and compared among the different stages of leukemia and lymphoma. Fold changes between the stages were calculated and those with statistical significance are presented in Table 2. In leukemia patients, mir-1 and mir-196a-5p were upregulated, particularly in the ND and Rem stages, compared to the Res stage. In lymphoma, mir-20a-5p, mir-23a-3p, and mir-92b-3p were upregulated with higher expression levels observed in the Res and ND stages compared to the Rem stage. The boxplots in Figure 2 illustrate the differential expression of the five miRNAs (hsa-miR-1, hsa-miR-20a-5p, hsa-miR-23a-3p, hsa-miR-92b-3p, and hsa-miR196a-5p) in different stages of each disease. The relationship between delta CT and expression of the miRNA is inversely correlated, i.e., a lower delta CT indicates higher expression of the miRNA at this stage.

### 3.5. Functional Enrichment Analysis for Target Genes

**Figure 3:**
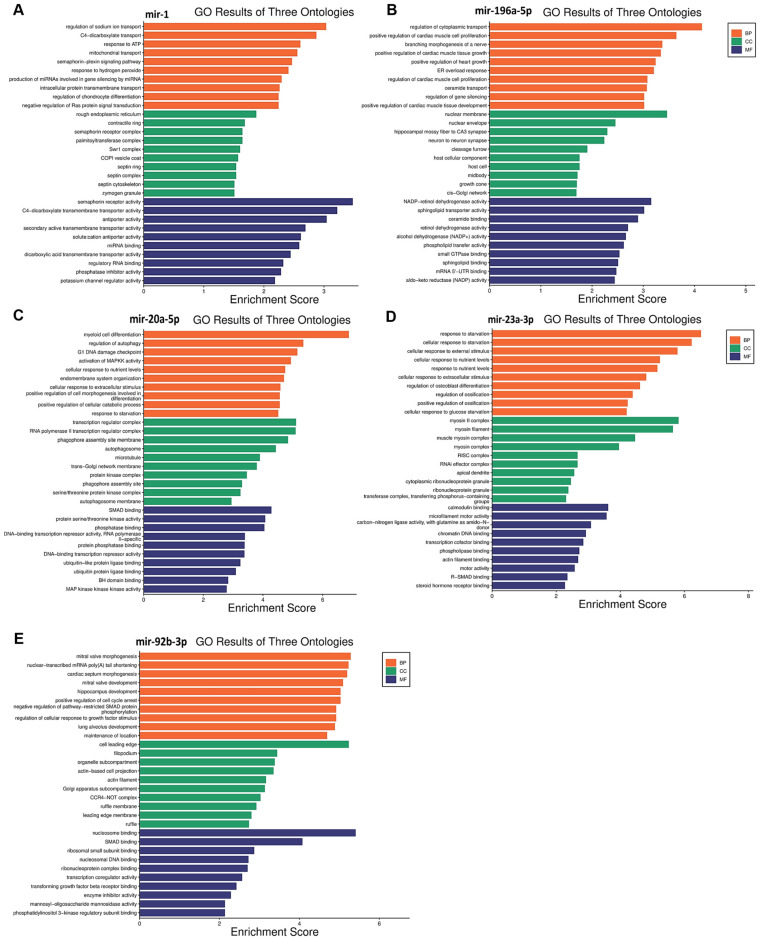
Gene ontology enrichment analysis results for five miRNAs in leukemia and lymphoma. (**A**) miR-1; (**B**) miR-196-5p; (**C**) miR-20a-5p; (**D**) miR-23a-3p; (**E**) miR-92b-3p. Biological process, cellular component, and molecular function terms are shown on the *Y*-axis, and the *X*-axis represents the fold enrichment score for each term.

**Figure 4:**
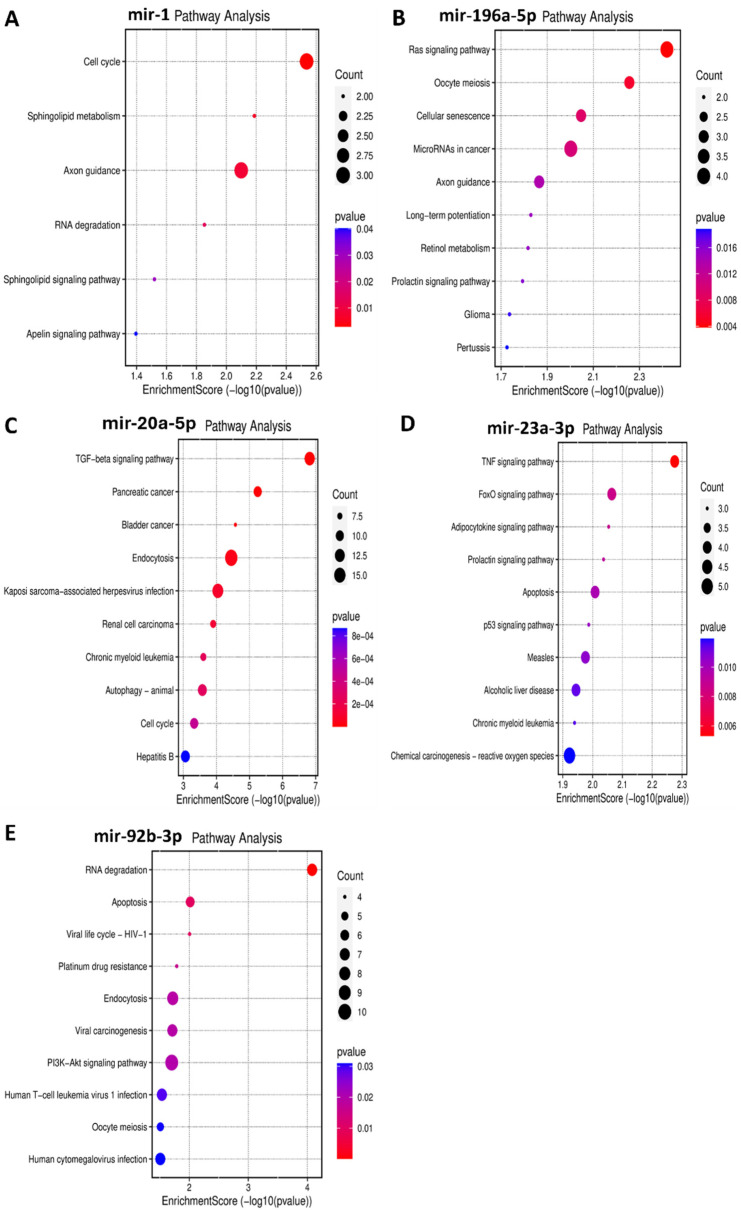
Bubble plots demonstrating enriched KEGG pathways for the five miRNAs in leukemia and in lymphoma. *Y*-axis represents the pathways that were associated with the miRNAs’ target genes, and the *X*-axis represents the fold enrichment score for each term. The bigger the size of the dot, the greater the degree of pathway enrichment. The red color indicates a lower chance of error, so higher confidence in the results. (**A**) miR-1 in leukemia significantly impacts the cell cycle, among other pathways. (**B**) miR-196a-5p in leukemia majorly impacts the RAS signaling pathway, among others. (**C**) miR-20a-5p in lymphoma significantly impacts the TGF–β signaling pathway, among others. (**D**) miR-23a-3p in lymphoma significantly impacts the TNF signaling pathway, among others. (**E**) miR-92b-3p in lymphoma significantly impacts the RNA degradation and apoptosis pathways, among others.

Functional enrichment analysis was performed for the target genes of the different miRNAs studied in this study to elucidate how their differential expression may impact cellular and molecular functions. A gene ontology analysis, shown in Figure 3, was performed to reveal three classes of biological outcomes for the gene sets, namely biological process (BP), cellular component (CC), and molecular function (MF). A detailed overview of the target genes for these five miRNAs is presented in Table 3. The enrichment analysis (Figure 4) revealed that hsa-miR1, hsa-miR-20a-5p, hsa-miR-23a-3p, hsa-miR-92b-3p, and hsa-miR-196a-5p are involved in important pathways in lymphoma and leukemia such as the RAS signaling pathway, the cell cycle, the TGF-beta signaling pathway, the TNF signaling pathway, RNA degradation, and in apoptosis.

## 4. Discussion

miRNAs are leading the field as essential clinical biomarkers for disease diagnosis of and prognosis due to their low complexity in comparison to proteins [44]. The differential expression of miRNAs at different stages of leukemia and lymphoma is still unclear. In this preliminary case–control study, a panel of 79 miRNAs were tested for their differential expression across three different stages (ND, Rem, and Res) in leukemia and lymphoma patients. Understanding the changes in their expression across these stages would not only aid in better diagnosis of the disease but will also allow for a better detection of disease progression and hence a better course of treatment.

The comparison between healthy controls and ND patients suggested that mir-223-3p was upregulated in the early stages of leukemia (Table 1). This is consistent with a previous study that reported its upregulation in T-cell acute lymphoblastic leukemia (T-ALL) compared to healthy controls due to the action of the Notch pathway and NF-κB, which are important in cancer pathogenesis [45]. In addition, mir-24-3p was upregulated in ND lymphoma patients (Table 1). A previous study showed that mir-24-3p was upregulated in Hodgkin lymphoma (HL) tissues, promoting cell invasion and proliferation [46]. This suggests that these miRNAs may be crucial in determining the disease pattern.

The qPCR analysis identified five miRNAs with significantly different expression levels in leukemia and lymphoma (Table 2): hsa-miR-1, hsa-miR-196a-5p, hsa-miR-20a-5p, hsa-miR-23a-3p, and hsa-miR-92b-3p. The fold change calculations indicated a significant upregulation of hsa-miR-1 and hsa-miR-196a-5p in leukemia and hsa-miR-20a-5p, hsa-miR-23a-3p, and hsa-miR-92b-3p in lymphoma.

hsa-miR-1 is a well-known tumor suppressor miRNA in several cancers like lung cancer, colorectal cancer, bladder cancer, prostate cancer, and others [47,48,49,50]. In this study, a significant upregulation of hsa-miR-1 was observed in patients with leukemia, specifically, hsa-miR-1 was higher in the Rem and ND stages compared to the Res stage and higher in the Rem group compared to the ND group. These results suggest that hsa-miR-1 could play an important role in disease progression and treatment. Hsa-miR-1 target genes are involved in RAS protein signal transduction and in crucial cancer pathways like semaphorin receptor signaling (Figure 3A). hsa-miR-1 downregulation of semaphorin receptor activity could limit the invasion of leukemia cells. Previously, semaphorin 4D was linked to the activation of the PI3K/AKT and ERK signaling pathways, which would lead to the development of leukemia cells [51]. Hence, the inhibition of semaphorin signaling pathways in leukemia may result in a better prognosis. These findings suggest that hsa-miR-1 upregulation in the Rem stage of leukemia could be correlated with a good prognosis. On the contrary, a study reported that the overexpression of miR-1 in AML cells increased disease aggressiveness in a mouse xenograft model in concordance with clinical data that reported poor patient survival [52]. This discrepancy might be due to multiple factors such as leukemia subtypes, genetic and epigenetic factors, and differences in study populations, experimental methodologies, therapeutic and environmental contexts, among others [53,54].

Another important upregulated miRNA in patients with leukemia in this study was mir-196a-5p, which was found to be highly expressed in the Rem stage compared to the Res stage and higher in the Rem stage compared to the ND stage. This finding suggests a potential role for miR-196a-5p in the disease pattern and prognosis of leukemia. Previous studies have implicated miR-196a-5p as an oncogene in several cancers including leukemia [55]. When compared to healthy bone marrow samples, miR-196a-1 was found to be significantly overexpressed in AML samples. However, in T-cell acute lymphoblastic leukemia (T-ALL), the expression of miR-196a-1 did not appear to have any predictive value [56]. These different reports demonstrate that the role of miR-196a-5p in leukemia is complicated and context-dependent.

Interestingly, the upregulation of miR-196a-5p observed in the Rem stage of leukemia in this study may suggest a favorable effect on the disease pattern. This is because the enrichment analysis (Figure 3B) revealed that miR-196a-5p plays a role in silencing the MAPKK pathway and small GTPase binding, which are important in the pathogenesis of leukemia. So, miR-196a-5p may help to maintain the Rem stage and inhibit disease progression.

However, the role of miR-196a-5p in leukemia is not entirely clear. According to a different study, overexpression of the closely related family member miR-196b was linked to increased proliferation and survival, and a partial block in bone marrow progenitor cell development in AML [57]. The different associations of miR-196a-5p with leukemia could be due to different subtypes of leukemia, different target populations, and the complex regulatory mechanisms underlying miRNA expression and function. Hence, further research and validation studies are needed.

mir-20a-5p is a component of the mir-17–92 cluster. It has been established that mir-20a-5p is strongly linked with cancer due to its oncogenic properties [58]. In this study, miR-20a-5p was significantly upregulated in lymphoma patients. Specifically, its levels were higher in the Res stage compared to the Rem stage and also higher in ND patients compared to both the Rem and Res stages. This finding suggests a potential role for miR-20a-5p in predicting the disease pattern and the prognosis of lymphoma. The expression levels of miR-20a-5p seem to affect several pathways, including downstream signaling pathways such as the TGF-β [59], MAPK [60], and PI3K/Akt pathways [61]. Interestingly, the upregulation of mir-20a-5p observed in the Res stage in patients with lymphoma in this study may suggest a poor prognosis with unfavorable events in the disease pattern. Several studies align with this logic, for example, elevated levels of miR-20a-5p in plasma have been seen in patients with diffuse large B-cell lymphoma (DLBCL), coupled with the dysregulation of other miRNAs. These findings suggest that miR-20a-5p may be a sensitive biomarker for lymphoma relapse as well as a marker for cancer diagnosis and prognosis [62]. Notably, miR-20a was also shown to activate the PTEN/PI3K/Akt signaling pathway in hepatocellular carcinoma, causing cells to become radiation resistant [63]. The enrichment analysis (Figure 3C) supports this data, showing that mir-20a-5p upregulation can inhibit apoptotic pathways, which helps cancer cells to survive and proliferate.

Another significantly upregulated miRNA in lymphoma patients in this study was miR-23a-3p. The expression of this miRNA was higher in the ND stage compared to the Res and Rem stages and also higher in the Res stage compared to the Rem stage. This upregulation suggests a possible involvement of this miRNA in the onset and progression of the disease. Prior studies have shown that miR-23a-3p and other oncogenic miRNAs, such as hsa-miR-24-3p, are overexpressed in classical Hodgkin lymphoma (cHL) cells [46,64]. The degree of tumor differentiation, the extent of metastasis, and the invasion of lymph nodes by malignancies are all correlated with the upregulation of this gene [65]. These results imply that miR-23a might have a role in the differentiation, metastasis, and staging of cancer. Hu et al. also reported that miR-23a expression enhanced gastric cancer growth and suppressed apoptosis [66]. Furthermore, miR-23a overexpression in pancreatic cancer was associated with poor survival [65], and hsa-miR-23a-3p expression in large B-cell lymphomas was associated with poor survival [67].

The enrichment analysis (Figure 3D) suggested that miR-23a-3p upregulation is linked to the inhibition of SMAD binding. This could disrupt TGF-β signaling, which is important for cell growth and differentiation. Thus, miR-23a-3p could be a target for therapeutic interventions in lymphoma as well as a prospective biomarker to monitor the advancement of the disease.

The last significantly upregulated miRNA in this study in patients with lymphoma was miR-92b-3p, which was higher in the Res stage compared to the ND and Rem stages, and higher in the ND stage compared to the Rem stage. This upregulation suggests an essential role for this miRNA in the pathogenesis of lymphoma. The miR-17~92 cluster, to which mir-92b-3p belongs, has been found to be often upregulated in human malignancies, including lymphomas. It is believed to drive lymphomagenesis by reducing negative regulators of oncogenic pathways, such as NF-κB and PI3K [68]. Moreover, the higher expression of miR-92b-3p in breast cancer was associated with larger tumors, increased lymph node metastases, and a poorer prognosis [69]. Additionally, in line with this study, elevated miR-92b-3p levels in small-cell lung cancer were found to target the PTEN/AKT pathway to enhance chemoresistance [70]. Taken together, it appears that the upregulation of mir-92b-3p in lymphoma could result in a poor prognosis. The enrichment analysis (Figure 3E) further supports this by showing that the upregulation of this miRNA could inhibit the expression of genes that play a role in apoptosis, such as those involved in SMAD binding, extrinsic and intrinsic apoptosis pathways, and the TORC2 complex, which are crucial for maintaining cellular balance and preventing cancer development. Consequently, the inhibition of these pathways by mir-92b-3p upregulation may enhance cell survival and growth, contributing to poor outcomes. Therefore, miR-92b-3p could serve as a target for therapeutic intervention in lymphoma as well as a prospective biomarker for monitoring the advancement of the disease.

The results of this preliminary study shed light on significant biomarkers in different stages of leukemia and lymphoma that need to be investigated further. However, there were some limitations to this study such as a small sample size, which was due to the limited number of available patients that lead to the absence of disease subtype analyses. Conducting the analyses on subtypes could potentially yield more significant results and provide a more detailed understanding of the disease pattern. As a follow-up, a proteomics study could provide more insight into how miRNAs and their target proteins participate in the pathogenesis of various leukemia and lymphoma subtypes. This in turn may lead to the development of novel personalized therapeutic approaches and new diagnostic markers.

## 5. Conclusions

The current study was carried out to identify the differential expression and functional implications of a panel of miRNAs in leukemia and lymphoma. The study highlighted the significant upregulation of five miRNAs (miR-1, miR-196a-5p, miR-20a-5p, miR-23a-3p, and miR-92b-3p) in the different stages of leukemia and lymphoma, suggesting that they have crucial roles in disease progression, treatment responses, and resistance mechanisms. The enrichment analysis revealed their involvement in essential cancer-related pathways, such as the RAS signaling, TGF- β signaling, and apoptotic pathways.

## Figures and Tables

**Table 1:** miRNA expression fold changes in newly diagnosed patients and healthy controls.

miRNA	Type of Disease	Stage and Fold Change		Trend
hsa-miR-223-3p	Leukemia	Healthy control	ND	Upregulation
		1	5.97	
hsa-miR-24-3p	Lymphoma	Healthy control	ND	Upregulation
		1	2.41	

**Table 2:** miRNA differential expression in ND, Rem, and Res stages in leukemia and lymphoma samples.

miRNA	*p*-Value	Disease Type	Stage and Fold Change Compared to ND Control			Trend	Potential Clinical Implications	Suggested Prognosis
hsa-miR-1	0.059	Leukemia	ND	Res	Rem	Up regulation	Higher in Rem and ND compared to Res, and higher in Rem compared to ND	Good
			1	0.38	2.43			
hsa-miR-196a-5p	0.059	Leukemia	ND	Res	Rem	Up regulation	Very high in Rem compared to Res, also higher in Rem compared to ND, and higher in ND compared to Res	Good
			1	0.51	1161.2			
hsa-miR-20a-5p	0.077	Lymphoma	ND	Res	Rem	Up regulation	Higher in Res compared to Rem, and higher in ND compared to Rem, and Res	Poor
			1	0.87	0.00			
hsa-miR-23a-3p	0.077	Lymphoma	ND	Res	Rem	Up regulation	Higher in ND compared to Res and Rem, but higher in Res compared to Rem	Poor
			1	0.44	0.01			
hsa-miR-92b-3p	0.064	Lymphoma	ND	Res	Rem	Up regulation	Higher in Res compared to ND and Rem, and higher in ND compared to Rem	Poor
			1	3.67	0.38			

**Table 3:** Differentially expressed miRNAs and their associated pathways in leukemia and lymphoma.

miRNA	Pathways	*p*-Value	Target Genes
hsa-miR-1	semaphorin receptor activity	0.0003	PLXNA4/MET
	RAS protein signal transduction	0.0390	G3BP1/RASA1/MET
hsa-miR-196a-5p	small GTPase binding	0.0029	RAB29/RCC2/EXOC8/RGL2/SMCR8/GGA3
	activation of MAPKK activity	0.0115	MAPK1/MAP3K21
hsa-miR-20a-5p	transforming growth factor beta receptor signaling pathway	0.0103	SPRED1/CREB1/CAV1/SMAD7/BAMBI/SKIL/SMAD5/SMAD4/SKI/ZBTB7A
	regulation of apoptotic signaling pathway	0.0281	DNM1L/ZNF385A/BCL2L2/BCL2L11/CAV1/SGMS1/MAPK9/SKIL/FEM1B/TRIM32/HIF1A/MCL1/RB1/RB1CC1/PLAGL2
hsa-miR-23a-3p	SMAD binding	0.0465	SMAD3/DDX5
	response to starvation	0.00003	TNRC6A/PPARGC1A/FNIP1/TBL2/FAS/FOXO3/KLF10/SESN2/ASNS
hsa-miR-92b-3p	extrinsic apoptotic signaling pathway	0.0001	FASLG/SGK3/DAB2IP/BCL2L11/MOAP1/ITGAV/BAK1/DDX3X/ITGA6/MCL1/SGPP1
	TORC2 complex	0.0093	SMG1/SESN3

## Data Availability

The datasets used and/or analyzed during the current study are available from the corresponding author on reasonable request.

## Supplementary Materials

The following supporting information can be downloaded at: https://www.mdpi.com/article/10.3390/biomedicines12081924/s1, Table S1: miRCURY LNA miRNA Focus PCR Panel with 96 miRNAs involved in tumorigenesis, apoptosis, and differentiation pathways (Qiagen miRCURY LNA miRNA Focus Panel—Cancer); Table S2: Patients Demographics; Table S3. miRNA differential expression in ND leukemia and lymphoma patients compared to healthy controls.

## List of Abbreviations

**Abbreviation**	**Description**
miRNAs	MicroRNA
HM	Hematological malignancy
AML	Acute myeloid leukemia
T-ALL	T-lymphoblastic leukemia
cDNA	Complementary DNA
MDS	Myelodysplastic syndrome
KAUH	King Abdullah University Hospital
IRB	Institutional Review Board
rpm	Revolutions per minute
μL	Microliter
CT	Cycle threshold
∆CT	Delta CT
GO	Gene ontology
KEGG	Kyoto Encyclopedia of Genes and Genomes
ND	Newly diagnosed
Rem	Remission
Res	Resistance
PI3K/AKT	Phosphatidylinositol 3-kinase/protein kinase B
ERK	Extracellular signal-regulated kinase
TORC2	Target of rapamycin complex 2
MAPKK	Mitogen-activated protein kinase kinase
cHL	Classical Hodgkin lymphoma
DLBCL	Diffuse large B-cell lymphoma
TGF-β	Transforming growth factor-β
Cat #	Catalogue number
