# Supplementary material for: miRNA Profiles in Patients with Hematological Malignancy at Different Stages of the Disease: A Preliminary Study
Source: Biomedicines. 2024 Aug 22;12(8):1924. doi: 10.3390/biomedicines12081924 (PMC11351647; doi:10.3390/biomedicines12081924)
Supplement: Supplementary file 1 [file biomedicines-12-01924-s001.zip › biomedicines-3137611-supplementary.pdf]

Supplementary Tables:

Table S1. miRCURY LNA miRNA Focus PCR Panel with 96-well miRNAs involved in tumorigenesis, apoptosis, and differentiation pathways (Qiagen miRCURY LNA miRNA Focus Panel – Cancer).

	1	2	3	4	5	6	7	8	9	10	11	12
A	hsa-let-7a-5p	hsa-let-7b-5p	hsa-let-7c-5p	hsa-let-7d-5p	UniSp2	hsa-miR-200a-3p	hsa-let-7g-5p	hsa-let-7i-5p	hsa-miR-1	hsa-miR-100-5p	hsa-let-7f-5p	hsa-miR-101-3p
B	hsa-miR-103a-3p	hsa-miR-106a-5p	hsa-miR-106b-5p	hsa-miR-107	UniSp4	UniSp6	hsa-miR-125b-5p	hsa-miR-126-3p	hsa-miR-130a-3p	hsa-miR-132-3p	hsa-miR-10b-5p	hsa-miR-133a-3p
C	hsa-miR-141-3p	hsa-miR-143-3p	hsa-miR-145-5p	hsa-miR-146a-5p	UniSp5	hsa-miR-26a-5p	hsa-miR-150-5p	hsa-miR-155-5p	hsa-miR-15a-5p	hsa-miR-15b-5p	hsa-miR-149-3p	hsa-miR-16-5p
D	hsa-miR-17-5p	hsa-miR-181a-5p	hsa-miR-181b-5p	hsa-miR-182-5p	cel-miR-39-3p	hsa-miR-27a-3p	hsa-miR-186-5p	hsa-miR-18a-5p	hsa-miR-191-5p	hsa-miR-192-5p	hsa-miR-148a-3p	hsa-miR-194-5p
E	hsa-miR-195-5p	hsa-miR-196a-5p	hsa-miR-19a-3p	hsa-miR-19b-3p	UniSp3 IPC	hsa-miR-200b-3p	hsa-miR-200c-3p	hsa-miR-202-3p	hsa-miR-10a-5p	hsa-miR-205-5p	hsa-miR-206	hsa-miR-20a-5p
F	hsa-miR-20b-5p	hsa-miR-21-5p	hsa-miR-210-3p	hsa-miR-214-3p	hsa-miR-215-5p	SNORD38B	hsa-miR-22-3p	hsa-miR-221-3p	hsa-miR-222-3p	hsa-miR-223-3p	U6 snRNA	hsa-miR-23a-3p
G	hsa-miR-23b-3p	hsa-miR-24-3p	hsa-miR-25-3p	UniSp3 IPC	hsa-miR-26b-5p	UniSp3 IPC	hsa-miR-27b-3p	hsa-miR-29a-3p	hsa-miR-29b-3p	hsa-miR-29c-3p	hsa-miR-30b-5p	hsa-miR-30c-5p
H	hsa-miR-30d-5p	hsa-miR-31-5p	hsa-miR-34a-5p	SNORD49A	hsa-miR-423-5p	hsa-miR-7-5p	hsa-miR-9-5p	hsa-let-7e-5p	hsa-miR-92b-3p	hsa-miR-93-5p	hsa-miR-99a-5p	Blank (H2O)

Table S2. Patients Demographics.

Gender	Type of HM	Age	Stage	Weight (kg)	Smoking	Treatment
F	CML	18	ND	62	No	None
F	CLL	29	ND	88	No	None
M	AML	49	ND	88	No	None
F	Non-Hodgkin Lymphoma	70	ND	106	No	None
F	Non-Hodgkin Lymphoma	26	ND	62	No	None
M	Hodgkin Lymphoma	28	ND	115	Yes	None
F	CLL	61	Rem	87	Yes	Chemotherapy
F	CML	66	Rem	146	No	Imatinib
F	CML	58	Rem	70	No	Imatinib
F	Hodgkin Lymphoma	27	Rem	70	No	Chemotherapy
F	Non-Hodgkin Lymphoma	38	Rem	70	No	Chemotherapy
M	Hodgkin Lymphoma	65	Rem	100	No	Chemotherapy
F	AML	37	Res	66	No	Chemotherapy
F	CLL	53	Res	53	No	Chemotherapy
M	CLL	54	Res	70	No	Chemotherapy
F	Hodgkin Lymphoma	65	Res	66	No	Chemotherapy
M	Non-Hodgkin Lymphoma	27	Res	70	Yes	Chemotherapy
M	Non-Hodgkin Lymphoma	46	Res	73	No	Chemotherapy
F	Healthy control	59	Healthy control	70	No	None
M	Healthy control	46	Healthy control	86	No	None

Table S3. miRNA differential expression in ND leukemia and lymphoma patients compared to healthy controls.

miRNAs	Disease type	Stages and fold change over control		Trend	Potential clinical implications
hsa-miR-19a-3p	Leukemia	Healthy control<br>1	ND<br>5.62	Up regulation	Higher in ND leukemia compared to healthy control
hsa-miR-19b-3p	Leukemia	Healthy control<br>1	ND<br>4.95	Up regulation	Higher in ND leukemia compared to healthy control
hsa-miR-20a-5p	Leukemia	Healthy control<br>1	ND<br>2.03	Up regulation	Higher in ND leukemia compared to healthy control
hsa-miR-223-3p	Leukemia	Healthy control<br>1	ND<br>5.97	Up regulation	Higher in ND leukemia compared to healthy control
hsa-miR-93-5p	Leukemia	Healthy control<br>1	ND<br>2.57	Up regulation	Higher in ND leukemia compared to healthy control
hsa-miR-106a-5p	Lymphoma	Healthy control<br>1	ND<br>2.85	Up regulation	Higher in ND lymphoma compared to healthy control
hsa-miR-107	Lymphoma	Healthy control<br>1	ND<br>2.36	Up regulation	Higher in ND lymphoma compared to healthy control
hsa-miR-126-3p	Lymphoma	Healthy control<br>1	ND<br>2.72	Up regulation	Higher in ND lymphoma compared to healthy control
hsa-miR-15a-5p	Lymphoma	Healthy control<br>1	ND<br>3.24	Up regulation	Higher in ND lymphoma compared to healthy control
hsa-miR-19a-3p	Lymphoma	Healthy control<br>1	ND<br>12.97	Up regulation	Higher in ND lymphoma compared to healthy control
hsa-miR-19b-3p	Lymphoma	Healthy control<br>1	ND<br>8.44	Up regulation	Higher in ND lymphoma compared to healthy control
hsa-miR-20a-5p	Lymphoma	Healthy control<br>1	ND<br>3.24	Up regulation	Higher in ND lymphoma compared to healthy control
hsa-miR-21-5p	Lymphoma	Healthy control<br>1	ND<br>15.41	Up regulation	Higher in ND lymphoma compared to healthy control
hsa-miR-223-3p	Lymphoma	Healthy control<br>1	ND<br>42.43	Up regulation	Higher in ND lymphoma compared to healthy control
hsa-miR-24-3p	Lymphoma	Healthy control<br>1	ND<br>2.41	Up regulation	Higher in ND lymphoma compared to healthy control
hsa-miR-93-5p	Lymphoma	Healthy control<br>1	ND<br>2.66	Up regulation	Higher in ND lymphoma compared to healthy control
